# Prediction of anti-vascular endothelial growth factor agent-specific treatment outcomes in neovascular age-related macular degeneration using a generative adversarial network

**DOI:** 10.1038/s41598-023-32398-7

**Published:** 2023-04-06

**Authors:** Sehwan Moon, Youngsuk Lee, Jeongyoung Hwang, Chul Gu Kim, Jong Woo Kim, Won Tae Yoon, Jae Hui Kim

**Affiliations:** 1 School of Electrical Engineering and Computer Science, Gwangju Institute of Science and Technology, Gwangju, South Korea; 2 INGRADIENT Inc., Seoul, South Korea; 3 AI Graduated School, Gwangju Institute of Science and Technology, Gwangju, South Korea; 4 MODULABS, Seoul, South Korea; 5 Department of Ophthalmology, Kim’s Eye Hospital, #156 Youngdeungpo-dong 4ga, Youngdeungpo-gu, Seoul, 150-034 South Korea; 6 Kim’s Eye Hospital Data Center, Seoul, South Korea

**Keywords:** Macular degeneration, Outcomes research, Machine learning

## Abstract

To develop an artificial intelligence (AI) model that predicts anti-vascular endothelial growth factor (VEGF) agent-specific anatomical treatment outcomes in neovascular age-related macular degeneration (AMD), thereby assisting clinicians in selecting the most suitable anti-VEGF agent for each patient. This retrospective study included patients diagnosed with neovascular AMD who received three loading injections of either ranibizumab or aflibercept. Training was performed using optical coherence tomography (OCT) images with an attention generative adversarial network (GAN) model. To test the performance of the AI model, the sensitivity and specificity to predict the presence of retinal fluid after treatment were calculated for the AI model, an experienced (Examiner 1), and a less experienced (Examiner 2) human examiners. A total of 1684 OCT images from 842 patients (419 treated with ranibizumab and 423 treated with aflibercept) were used as the training set. Testing was performed using images from 98 patients. In patients treated with ranibizumab, the sensitivity and specificity, respectively, were 0.615 and 0.667 for the AI model, 0.385 and 0.861 for Examiner 1, and 0.231 and 0.806 for Examiner 2. In patients treated with aflibercept, the sensitivity and specificity, respectively, were 0.857 and 0.881 for the AI model, 0.429 and 0.976 for Examiner 1, and 0.429 and 0.857 for Examiner 2. In 18.5% of cases, the fluid status of synthetic posttreatment images differed between ranibizumab and aflibercept. The AI model using GAN might predict anti-VEGF agent-specific short-term treatment outcomes with relatively higher sensitivity than human examiners. Additionally, there was a difference in the efficacy in fluid resolution between the anti-VEGF agents. These results suggest the potential of AI in personalized medicine for patients with neovascular AMD.

## Introduction

Neovascular age-related macular degeneration (AMD) is a vision-threatening disorder that can lead to severe visual loss^1^. With the acceleration of global aging, the prevalence of AMD is anticipated to increase as well^2^, highlighting the importance of treatment for neovascular AMD in the future.

Anti-vascular endothelial growth factor (VEGF) therapy is currently a mainstay treatment for neovascular AMD^3^. The first widely-used, Food and Drug Administration (FDA) approved anti-VEGF agent was ranibizumab^4^, followed by aflibercept^5^. More recently, brolucizumab^6^ and faricimab^7^ also gained FDA approval to treat neovascular AMD. Although all FDA-approved anti-VEGF agents are effective in treating neovascular AMD, the efficacy between different agents differs^8–11^.

In particular, the difference occasionally appears immediately after the initial loading injections^11–14^. Since the initial treatment outcome is closely associated with long-term prognosis^15^, an appropriate anti-VEGF agent should be selected for patients, which might lead to good initial treatment outcomes. Moreover, since anti-VEGF agents are generally expensive^16^, selecting one that can completely resolve retinal fluid in the initial treatment may help reduce the patient’s financial burden. To date, accurate determination of the most suitable anti-VEGF agent for patients is challenging.

Recently, several investigators focused on artificial intelligence (AI)-based prediction of treatment outcomes in neovascular AMD^17–21^. Although their AI models provided reliable outcomes, the potential difference in the outcomes among the different anti-VEGF agents was not evaluated. AI-assisted personalized medicine is one of the current major issues in the field of medicine^22,23^. In the treatment of neovascular AMD, the first step for personalized medicine is to select the appropriate anti-VEGF agent for the patient. Predicting treatment outcomes specific to anti-VEGF agents can be challenging for doctors. Moreover, to our knowledge, no prior studies have focused on this particular topic. Therefore, it would be highly beneficial if AI could assist in this prediction. In the treatment of neovascular AMD, optical coherence tomography (OCT) is widely utilized and is likely the most important diagnostic tool in clinical practice. Therefore, predicting post-treatment OCT images may hold significant value for clinical practice, as well as research purposes.

The present study aimed to establish an AI model using a generative adversarial network (GAN) to predict short-term anatomical outcomes in neovascular AMD and to evaluate whether the model could differentiate between the outcomes of ranibizumab and aflibercept.

## Materials and methods

This retrospective observational study was conducted at a single center: Kim’s Eye Hospital, Seoul, South Korea. The study was approved by the Institutional Review Board (IRB) of Kim’s Eye Hospital and was conducted in accordance with the tenets of the Declaration of Helsinki. Owing to the retrospective nature of this study, the need for informed consent was waived by Kim’s Eye Hospital IRB.

### Study participants and treatment

This study included treatment-naïve patients who were diagnosed with type 3 macular neovascularization (MNV) between January 2013 and December 2018, and who were initially treated with three loading injections of an anti-VEGF. The exclusion criteria were as follows: (1) less than 3 months of follow-up, (2) history of vitreoretinal surgery or glaucoma surgery, and (3) low OCT image quality, such as severe noise hindering AI learning. When both eyes of the patients met the inclusion criteria, those with prior symptoms were enrolled in the study.

At initial diagnosis, all patients underwent fluorescein angiography and OCT. All OCT scans were performed using a Spectralis device (Heidelberg Engineering, Heidelberg, Germany). To obtain high-quality OCT images and minimize noise, the Automatic Real Time-function (ART) technique was used; at least 80 scans were averaged to generate each OCT image. All patients underwent three monthly injections of ranibizumab (0.5 mg/0.05 mL of Lucentis; Genentech Inc., San Francisco, CA, USA) or aflibercept (2.0 mg/0.05 mL of Eylea; Regeneron, Tarrytown, NY, USA). One month after the third injection, OCT was repeated.

### AI model learning and image synthesis

Horizontal and vertical OCT crosshair scan images taken before treatment and after the three loading injections were used for AI model learning. There was no other special image processing for AI learning. The original images, initially sized 1000 × 1000 pixels, were resized to 256 × 256 pixels. To compensate for pixel-wise intensity differences between pre-therapeutic and post-therapeutic images that might affect model training, registration was performed using the affine registration toolbox in SimpleElastix.

**Figure 1:**
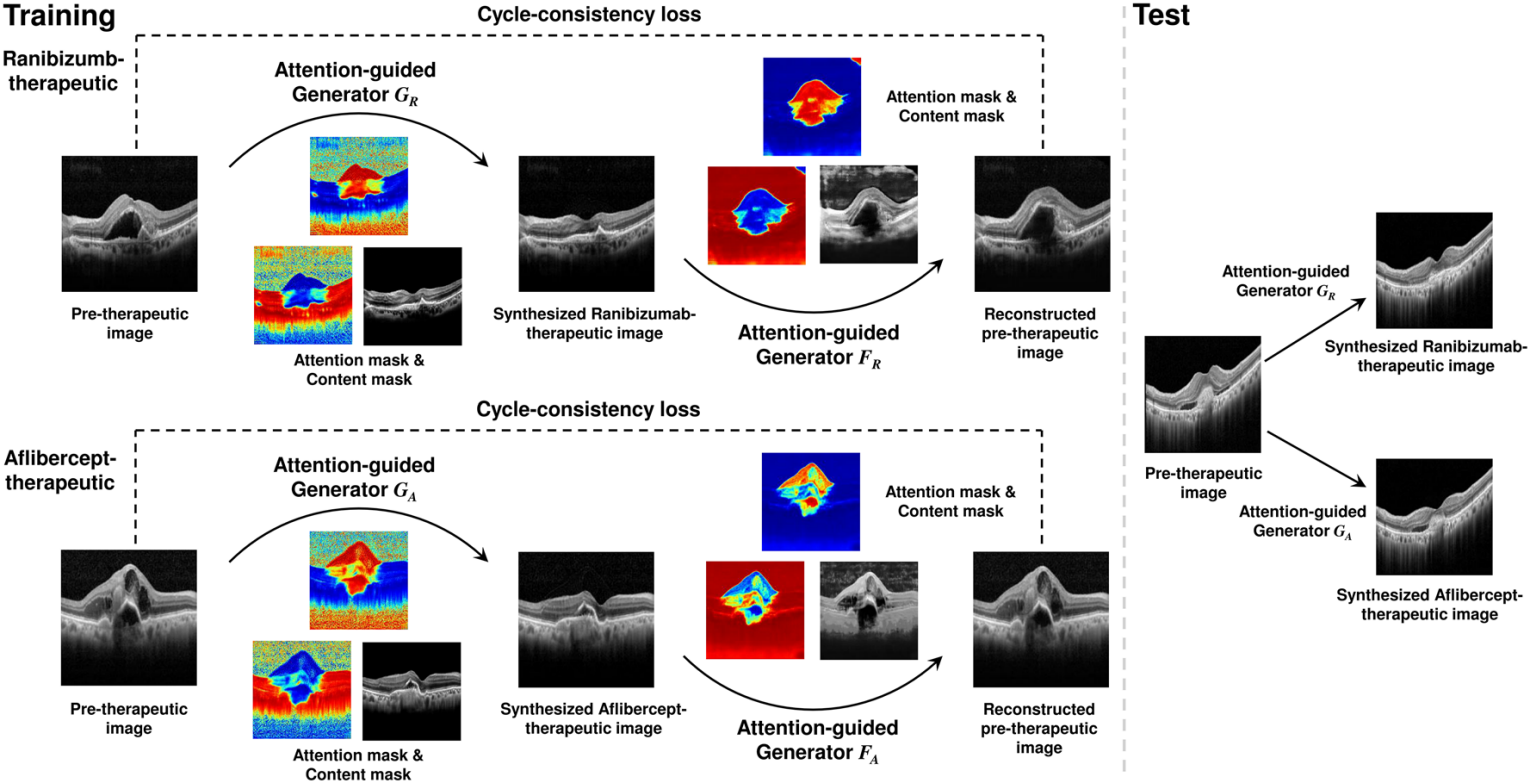
Overview of the proposed post-therapeutic optical coherence tomography image synthesis model. Models are trained with a training set of each anti-vascular endothelial growth factor agent. For ranibizumab, the model consists of two attention-guided generators, G-ranibizumab and F-ranibizumab. For aflibercept, the model consists of G-aflibercept and F-aflibercept. Each generator aims to detect the discriminative pixel of the image. After that, the input image, content mask, and attention mask are mixed to manufacture the synthesized therapeutic image or reconstructed pre-therapeutic image. Moreover, the models are constrained by cycle-consistency loss.

The AttentionGAN algorithm was adopted in post-therapeutic OCT image synthesis^24^, where the AI model focused on changes in the area of the foreground when producing post-therapeutic OCT images. The background image was maintained. We constructed a model based on AttentionGAN to identify differences in treatment outcomes according to different anti-VEGF agents (Fig. 1). The AI model did not separately identify or learn pathologic findings such as subretinal fluid (SRF) or intraretinal fluid (IRF).

To synthesize post-OCT images according to the anti-VEGF agents, generative models were separately trained for each anti-VEGF agent; it is suitable for tasks with high overlapping similarity between the input and output images. The attention-guided generator computes the content, foreground, and background attention masks. A content mask is an intermediate bridge for generating post-therapeutic OCT images. The foreground attention mask defines the partial contribution of the content mask, and the background attention mask preserves the background of a given OCT image. The images generated through the content and foreground attention masks and those through the background attention mask were fused. This makes the post-therapeutic OCT more realistic. The generated post-treatment OCT images were used to train another attention generator to reconstruct pre-treatment OCT images. The two discriminators were trained to classify whether the generated post-therapeutic and reconstructed pre-therapeutic OCT images were real. With the cycle-consistency loss, the reconstructed pre-therapeutic OCT and generated post-therapeutic OCT images were trained to be similar to each real image.

All experiments were performed using the PyTorch deep learning framework (ver. 1.12.0) in Python (ver. 3.7.4) (Python Software Foundation, Wilmington, DE, USA) using an NVIDIA Tesla V100 (NVIDIA, Santa Clara, CA, USA). We applied the default hyperparameters used in a previous AttentionGAN study^24^, except for three convolutional layer filter sizes.

### Comparison between the AI model and human retina specialists

Testing of the performance of the AI model was planned using horizontal and vertical cross-hair OCT images from 100 patients (50 treated with ranibizumab and 50 with aflibercept) who were not included in the AI model training. Images obtained before and after the three loading injections were analyzed. Among these, non-interpretable synthetic images of poor quality were excluded. The performance of the AI model was compared with that of experienced (J.H.K.; Examiner 1) and less experienced (W.T.Y.; Examiner 2) retina specialists. Residual retinal fluid was considered to be present when SRF or IRF was noted on either horizontal or vertical OCT images. The sensitivity, specificity, and accuracy of predicting the presence of residual retinal fluid after treatment were calculated.

### Additional analysis

The logarithm of minimal angle of resolution (logMAR) best-corrected visual acuity (BCVA) and central retinal thickness (CRT) before and after, as well as the incidence of residual fluid after the three loading injections was compared between the ranibizumab and the aflibercept groups. Data from patients included in the testing set was used in this analysis.

### Statistical analysis

Data are presented as the mean ± standard deviation or number (percentage, %), where applicable. Differences in characteristics between the training and testing sets were compared using an independent sample t-test or chi-square test. The independent sample t-test and chi-square test were used for the comparison between the ranibizumab and aflibercept groups. In these analyses, Statistical Package for Social Sciences for Windows (version 21.0; IBM, Armonk, NY, USA) was used, and statistical significance was set at *p* < 0.05.

## Results

A total of 1684 OCT images from 842 patients were included in the training set (Table 1).

**Table 1:** Characteristics of patients included in the training and testing sets.

Characteristics	Training set (n = 842)	Testing set (n = 98)	*p*-value
Age	70.17 ± 8.4	72.41 ± 8.8	0.013*
Sex			0.318^†^
Men	506 (60.1%)	64 (65.3%)	
Women	336 (39.9%)	34 (34.7%)	
Anti-VEGF agent			0.965^†^
Ranibizumab	419 (49.8%)	49 (50%)	
Aflibercept	423 (50.2%)	49 (50%)	
Retinal fluid compartment before treatment			
Subretinal fluid	733 (87.1%)	87 (88.8%)	0.423^†^
Intraretinal fluid	294 (34.9%)	37 (37.8%)	0.400^†^
Complete fluid resolution after treatment	647 (76.8%)	79 (80.6%)	0.578^†^

Data are presented as mean ± standard deviation or percentage (%), where applicable.

VEGF, vascular endothelial growth factor.

*Independent sample t-test.

^†^Chi-square test.

The mean age was 70.2 ± 8.4 years. A total of 419 (49.8%) patients were treated with ranibizumab and 423 (50.2%) with aflibercept. When comparing the training and testing sets, there was no difference in sex (*p* = 0.318), the anti-VEGF agent used (*p* = 0.965), presence of SRF (*p* = 0.423) or IRF (*p* = 0.400) before treatment, and complete fluid resolution after treatment (*p* = 578). The patients included in the testing set were older than those included in the training set (*p* = 0.013).

### Performance of the AI model in predicting post-treatment OCT images

Among the images originally selected to evaluate the performance of the AI model in predicting post-treatment OCT images, those obtained from two patients were excluded because the quality of the synthesized images was considered non-interpretable. The performance of the AI model was tested based on images from 98 patients (49 treated with ranibizumab and 49 with aflibercept) (Table 1).

Table 2 summarizes the sensitivity, specificity, and accuracy in predicting the presence of residual fluid after treatment.

**Table 2:** Sensitivity, specificity, and accuracy of the artificial intelligence (AI) model and the examiners in predicting the presence of residual retinal fluid after treatment.

Characteristics	AI model	Examiner 1	Examiner 2
Ranibizumab group (n = 49)			
Sensitivity	0.615	0.385	0.231
Specificity	0.667	0.861	0.806
Accuracy	0.653	0.735	0.653
Precision	0.400	0.500	0.300
Recall	0.615	0.385	0.231
Aflibercept group (n = 49)			
Sensitivity	0.857	0.429	0.429
Specificity	0.881	0.976	0.857
Accuracy	0.878	0.898	0.796
Precision	0.545	0.750	0.333
Recall	0.857	0.429	0.429
Retinal fluid compartment (n = 98)*			
Subretinal fluid		N/A	N/A
Sensitivity	0.500		
Specificity	0.837		
Accuracy	0.760		
Precision	0.300		
Recall	0.500		
Intraretinal fluid		N/A	N/A
Sensitivity	0.455		
Specificity	0.897		
Accuracy	0.847		
Precision	0.357		
Recall	0.455		

Data are presented as percentage (%).

*Evaluation of the retinal fluid compartment was performed only for the AI model.

N/A, not applicable.

**Figure 2:**
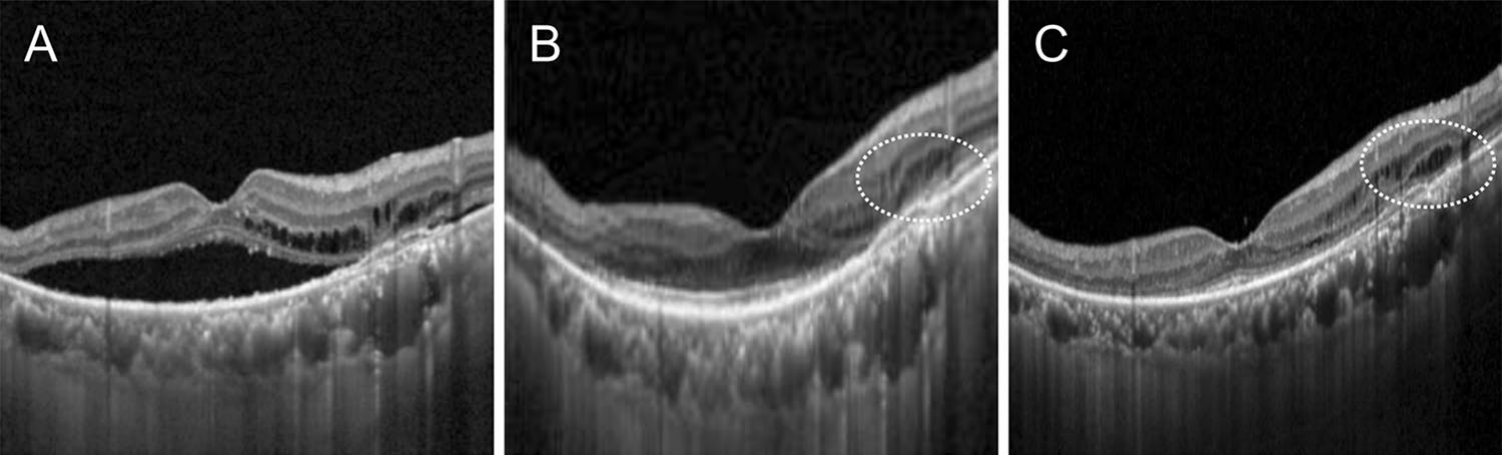
A representative case showing the difference in the prediction of post-treatment retinal fluid status between human retina specialists and artificial intelligence (AI) models. Before treatment, both subretinal fluid and intraretinal fluid (IRF) are noted on an optical coherence tomography image (**A**). Two human examiners predicted the complete resolution of retinal fluid after aflibercept treatment, whereas the AI model predicted the remaining IRF (**B**, synthetic image, dotted circle). Residual IRF is noted (dotted circle) as per the AI model prediction on a real image obtained after aflibercept treatment (**C**).

In general, the AI model showed a relatively higher sensitivity than the human examiners (Fig. 2).

**Figure 3:**
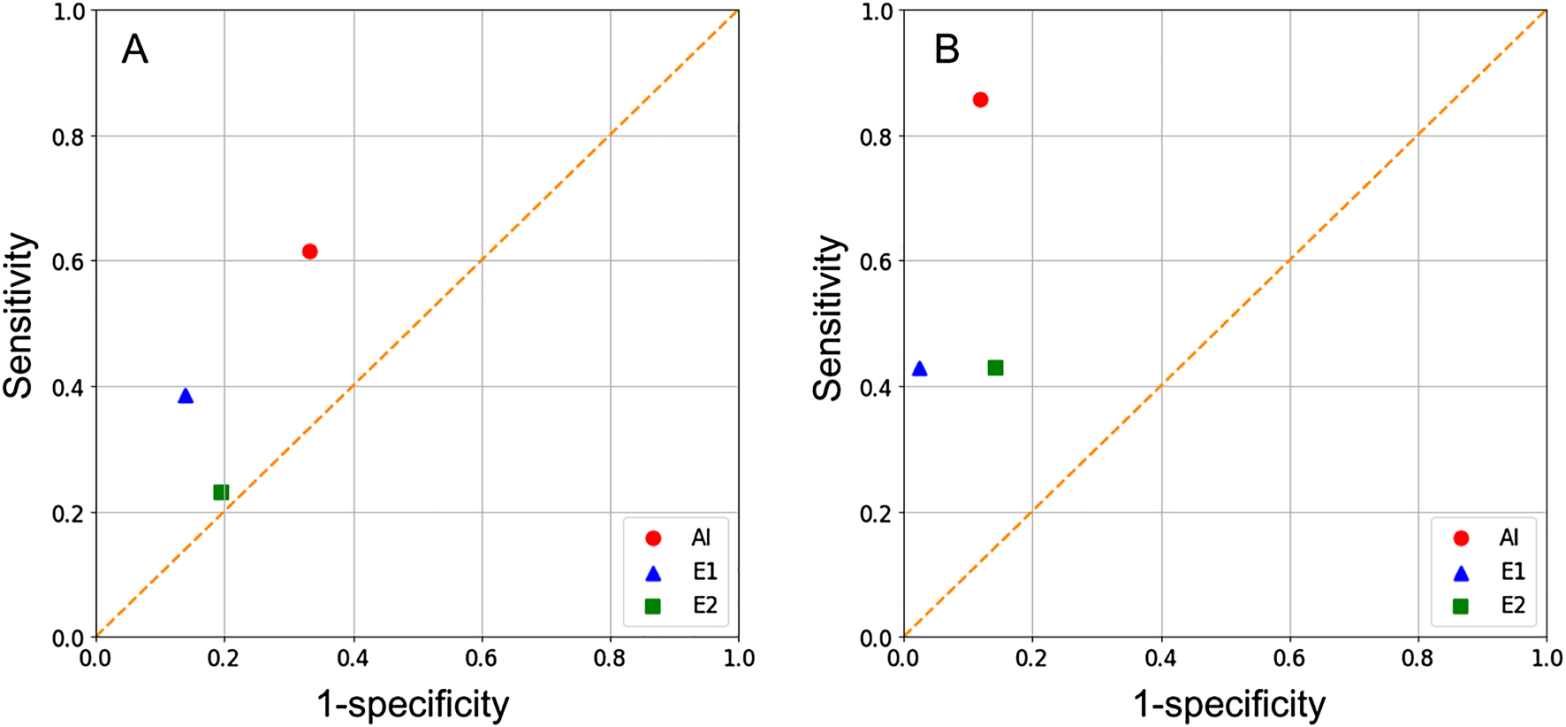
Comparison of the performance between the artificial intelligence model (AI), experienced human examiner (E1), and less experienced human examiner (E2) in predicting residual retinal fluid after initial treatment. In the ranibizumab group (**A**, n = 49), the sensitivity and specificity of the AI model are noted to be 0.615 and 0.667, respectively. In the aflibercept group (**B**, n = 49), the values are 0.857 and 0.881, respectively. Overall, the AI model shows comparable or relatively superior sensitivity and specificity than the human examiners.

In patients treated with ranibizumab (n = 49), the sensitivity and specificity, respectively, were 0.615 and 0.667 for the AI model, 0.385 and 0.861 for Examiner 1, and 0.231 and 0.806 for Examiner 2 (Fig. 3).

**Figure 4:**
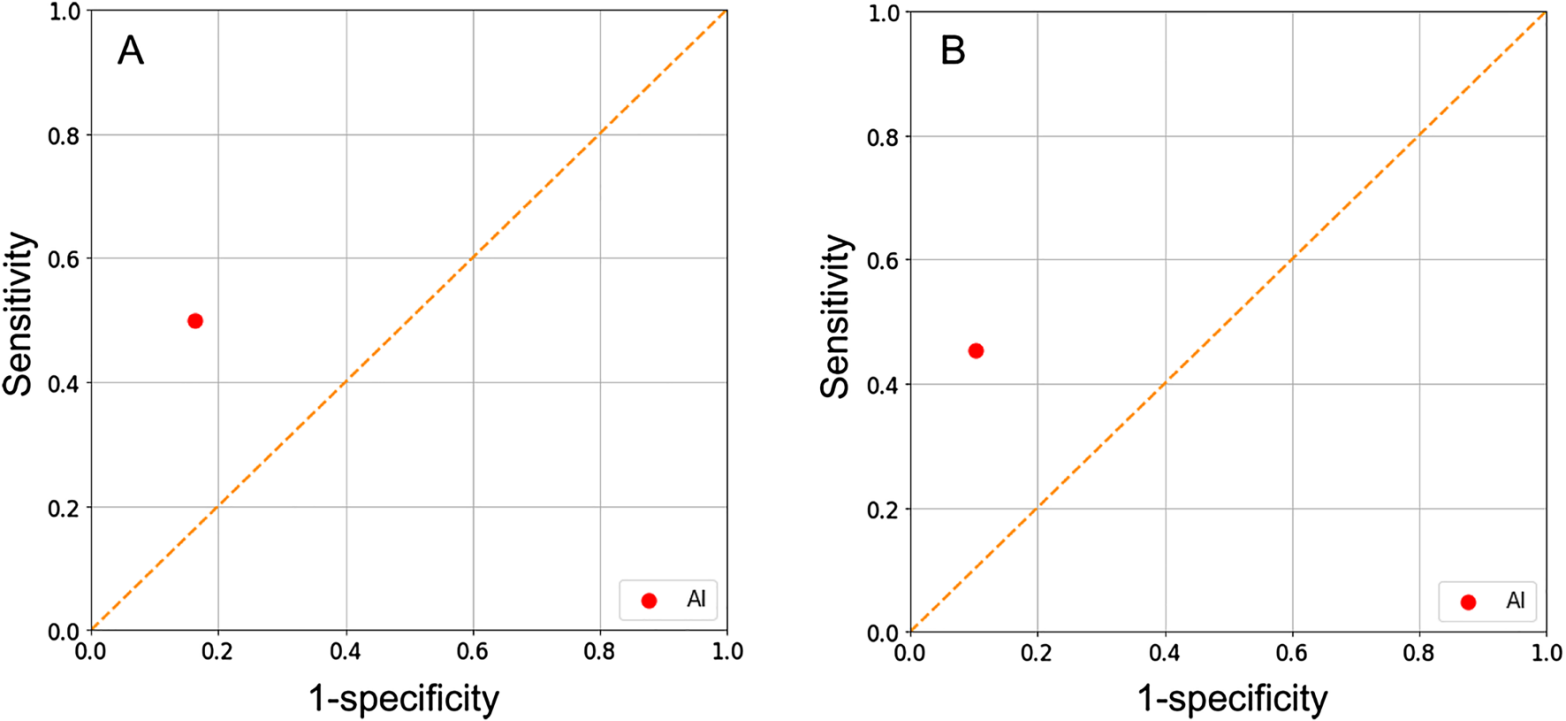
Performance of the artificial intelligence model in predicting residual subretinal fluid (**A**) and intraretinal fluid (**B**) after three loading injections of either ranibizumab or aflibercept (n = 98). The sensitivity and specificity, are noted to be 0.500 and 0.837 for SRF and 0.455 and 0.897 for IRF, respectively. The performance of the human examiners was not evaluated in this analysis.

In patients treated with aflibercept (n = 49), the sensitivity and specificity, respectively, were 0.857 and 0.881 for the AI model, 0.429 and 0.976, for Examiner 1, and 0.429 and 0.857, respectively, for Examiner 2 (Fig. 3). In all 98 patients, the sensitivity and specificity of the AI model in predicting the presence of SRF after treatment were 0.500 and 0.837, respectively (Fig. 4). The IRF values were 0.455 and 0.897, respectively (Fig. 4).

**Figure 5:**
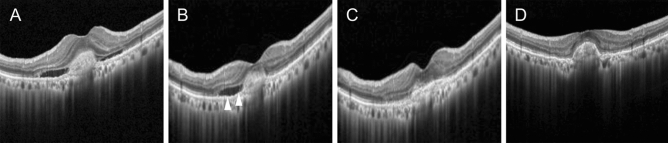
A representative case showing differences in the prediction of post-treatment retinal fluid status, according to the type of anti-vascular endothelial growth factor agent. (**A**) Subretinal fluid (SRF) is noted on the optical coherence tomography image obtained before treatment. Residual SRF is noted (arrowheads) on the artificial intelligence (AI)-synthetic image after ranibizumab treatment (**B**), whereas the complete resolution of retinal fluid is noted on the AI-synthetic image after aflibercept treatment (**C**). Complete resolution of retinal fluid is noted on a real image after aflibercept treatment (**D**).

In 18 (18.4%) of 98 patients, there was a difference in the fluid status of the AI-predicted post-treatment synthetic images between ranibizumab and aflibercept (Fig. 5).

**Figure 6:**
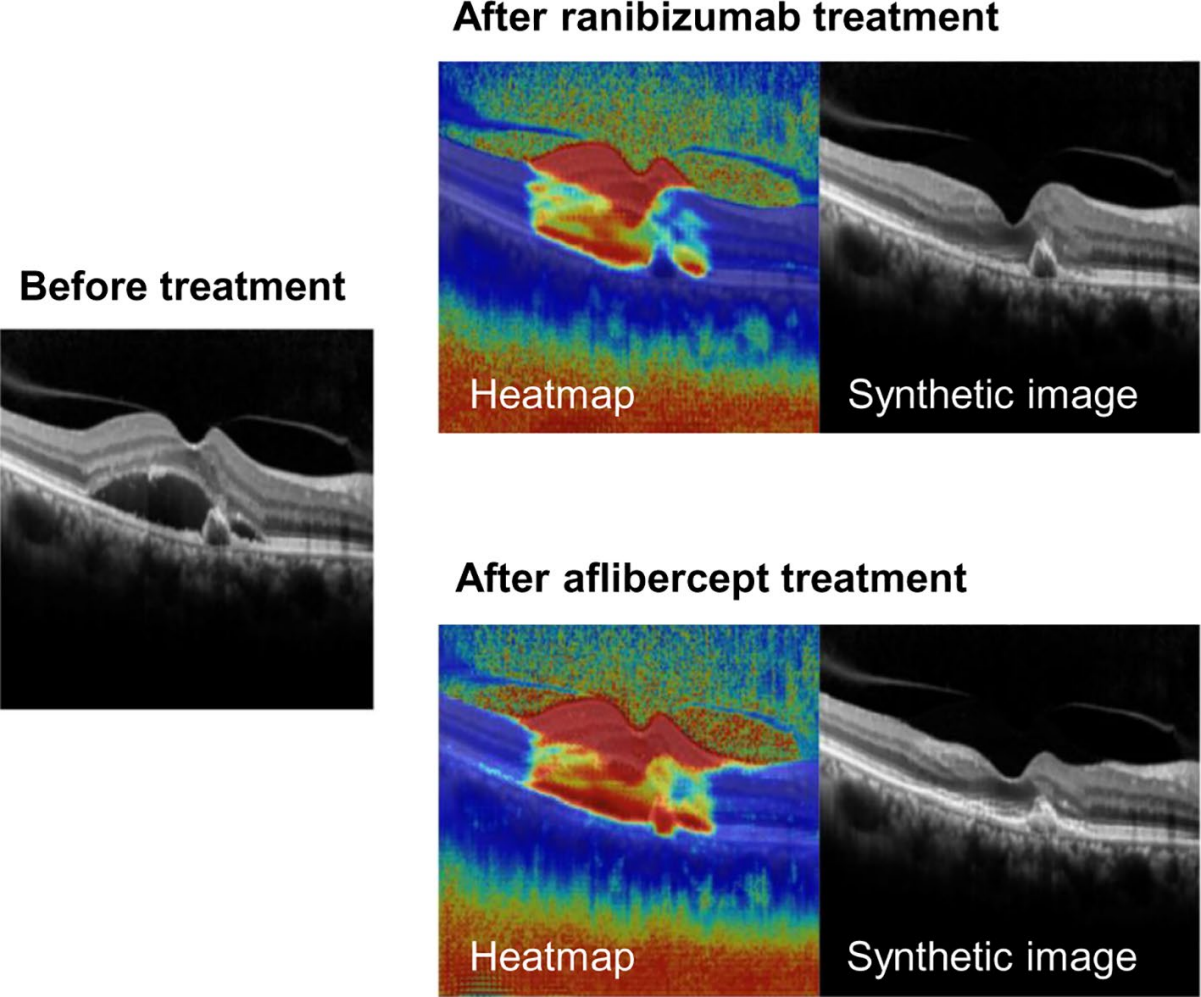
A representative figure of heatmap images shows that the artificial intelligence model identifies pathologic lesions of the retina. Additionally, there is a difference in heatmap images between ranibizumab and aflibercept treatments.

More specifically, the remaining fluid was predicted on post-treatment synthetic OCT images after ranibizumab treatment, but not after aflibercept treatment in these patients. Figure 6 shows the difference between the heatmap images of ranibizumab and aflibercept treatments, suggesting a potential difference in the AI algorithm in post-treatment image synthesis.

In comparison between the ranibizumab (n = 49) and aflibercept (n = 49) groups, there was no significant difference in the BCVA before (0.79 ± 0.55 vs. 0.66 ± 0.39; *p* = 0.170) and after (0.66 ± 0.57 vs. 0.47 ± 0.49; *p* = 0.086) the three loading injections. In addition, there was no difference in the CRT before (457.4 ± 158.1 vs. 469.7 ± 134.9; *p* = 0.679) and after (298.2 ± 140.2 vs. 291.8 ± 93.0; *p* = 0.786) the three loading injections, or the incidence of residual fluid after the injections (24.5% vs. 14.3%; *p* = 0.201).

## Discussion

To date, various AI methods have been used to predict the response of patients with neovascular AMD to anti-VEGF therapy. The two main arms of these studies were predicting post-treatment visual outcome^17,18^ and predicting post-treatment OCT features^19–21^. Rohm et al. attempted to predict visual acuity (VA) at 3 and 12 months using machine learning^17^. For the 3-month VA forecast, the difference between the prediction and ground truth was between 5.5 and 9 letters of mean absolute error. More recently, Yeh et al. evaluated the accuracy of a novel convolutional neural network in predicting 12-month visual outcomes in neovascular AMD^18^. The accuracy was reported to be 0.936.

Liu et al. predicted OCT images after anti-VEGF treatment for neovascular AMD using GAN^19^. In their study, 92% of the synthetic OCT images were of sufficient quality for clinical interpretation. The accuracy of predicting the macular status as wet or dry was 0.85. Zhao et al. attempted to predict the short-term anti-VEGF treatment responder/non-responder for neovascular AMD based on OCT images^21^. They used a novel sensitive structure-guided network, and the accuracy to predict the response was 84.6%. This accuracy was relatively better than that of deep learning-based methods as well as that of experienced ophthalmologists^21^. In the study by Lee et al., post-treatment OCT images were predicted using GAN. The accuracy was improved to 80.7–96.3% by the addition of baseline fluorescein angiography and indocyanine green angiography images^20^.

### The performance of the AI model in predicting post-treatment OCT images

In the present study, the AI model was established based on AttentionGAN, which has the advantage of creating post-therapeutic OCT images by concentrating the treatment area while maintaining the unchanging background. Additionally, the cycle-consistency loss provided by AttentionGAN generates more realistic post-therapeutic OCT images.

In general, the AI model showed a relatively superior performance to the examiners in predicting post-treatment fluid status. In particular, the sensitivity of the AI model was markedly higher than that of the examiners, suggesting that the AI model performed well in predicting “presence of residual fluid” after treatment. Contrastingly, the performance of the AI model was similar to or relatively lower than that of the experienced human examiner in predicting “complete resolution of retinal fluid” after treatment.

### Importance of predicting the difference in post-treatment OCT images between different anti-VEGF agents in personalized medicine

Similar to previous studies^19–21^, we attempted to generate post-treatment OCT images by using AI. The novel aspect of the present study is that we focused on the differences in post-treatment fluid status between patients treated with different anti-VEGF agents. The selection of an anti-VEGF agent is one of the key early steps in the treatment of neovascular AMD. One of the important factors predictive of long-term treatment outcomes is the fluid status after initial loading injections^25–27^. More specifically, patients exhibiting retinal fluid after initial loading injection were more likely to show poor long-term visual outcomes^25,27^ and required more intensive long-term injections^26^ than those not exhibiting. Therefore, it is important to select an agent that will result in the complete resolution of the retinal fluid through the initial loading injection.

Although both ranibizumab and aflibercept are effective, some differences in efficacy exist between the two. More specifically, aflibercept showed slightly superior efficacy in terms of anatomical improvement. In polypoidal choroidal vasculopathy, a higher rate of resolution of polypoidal lesions and greater decrease in retinal thickness were observed after aflibercept therapy than that after ranibizumab therapy^12,28^. Additionally, switching from ranibizumab to aflibercept may be beneficial in some patients with refractory neovascular AMD^29^. These findings suggest that aflibercept may be advantageous over ranibizumab in fluid resolution after initial loading injections.

However, aflibercept has several potential drawbacks. A part of the intravitreally injected anti-VEGF agent enters the systemic circulation, resulting in a reduction in systemic VEGF levels^30^. It is well known that aflibercept induces a more profound reduction in systemic VEGF levels than ranibizumab^30^. There is controversy as to whether this difference actually leads to a clinically significant impact^31^. However, several investigators have raised concerns regarding the potential side effects of VEGF reduction, especially in elderly patients^32–34^. In fact, several studies have suggested some impact of this concern in clinical practice^35,36^.

Personalized medicine refers to customized treatment for an individual patient based on the characteristics of the patient and the disease. When considering safety in elderly patients with neovascular AMD, ranibizumab may be prioritized as a treatment option for its favorable systemic risk profile. However, if ranibizumab treatment is not expected to be sufficiently effective in a patient, aflibercept may also be considered. In making such choices, it is necessary to involve as detailed a prediction as possible regarding the response to treatment. Although several biomarkers associated with response to anti-VEGF therapy have been elucidated^37^, there is no established biomarker to predict the difference in efficacy between ranibizumab and aflibercept. We believe that our AI algorithm may help predict the efficacy of the two agents and subsequently contribute to the selection of appropriate anti-VEGF agents for patients.

### Prediction of changes in each fluid compartment after treatment

Traditionally, the presence of retinal fluid in neovascular AMD has been considered an indicator of disease activity. Thus, fluid resolution is one of the primary targets of anti-VEGF therapy^38^. However, recent investigations have elucidated the different influences of each fluid compartment on visual outcomes^39^. For example, IRF is consistently associated with an unfavorable visual prognosis, unlike SRF^39^. Thus, each fluid compartment should be considered separately when determining the treatment strategy.

In the present study, we first attempted to predict the post-treatment status of each fluid compartment using the AI algorithm. Consequently, the performance of the AI algorithm was similar in predicting the residual SRF and IRF. We anticipate that further training of the algorithm using larger image sets will improve the performance of the AI-based prediction of each fluid compartment status. Furthermore, if this improved AI algorithm can be combined with automated detection of each retinal fluid compartment^40^, it may contribute to the establishment of a better AI-based patient follow-up system^41^.

### Utilization of post-treatment OCT image prediction in the application of newly introduced agents

The AI-based prediction of post-treatment OCT images using pre-existing agents is also useful in selecting candidates for administering newly introduced agents to treat neovascular AMD. Drug development for neovascular AMD is ongoing. In 2019, a new VEGF-A blocking agent brolucizumab^6^ was introduced. In 2022, faricimab^7^, which blocks VEGF-A and angiopoietin-2, was approved by the FDA. Currently, novel agents that improve the treatment outcomes of neovascular AMD, such as OPT-302^42^ and aflibercept 8.0 mg, are under development.

Newly introduced agents usually show similar or even superior outcomes compared to pre-existing agents^6,7^. However, in some instances, unexpected adverse events may occur when using new agents^43^. Thus, some doctors may take a conservative approach to using new drugs if the effect of pre-existing drugs is considered sufficient to treat the disease. However, if the effect of pre-existing drugs is not sufficient, utilizing a new drug would be a useful treatment option. Currently, ranibizumab and aflibercept are the two most widely used FDA-approved agents for treating neovascular AMD. Therefore, our algorithm, which can predict the responses of the two agents, will be of great assistance in identifying appropriate candidates for new agents.

In the past, the primary focus of AI utilization in the field of ophthalmology was the recognition of abnormal findings and diagnosis of diseases^44^. AI has been increasingly applied in other fields of medicine such as personalized medicine, including treatment selection and dose determination^45,46^. This trend is expected to be followed in ophthalmology. We anticipate that our study will contribute to the development of AI-based personalized medicine in ophthalmology.

The strength of the present study is that we first attempted to predict the difference in post-treatment OCT images according to the type of anti-VEGF. Additionally, the ability of the AI algorithm to predict the changes in each fluid compartment was analyzed. However, this study has several limitations. First, it was a retrospective study performed at a single center. Second, only the short-term outcomes were evaluated. Third, because only horizontal and vertical OCT scan images were used for analysis, the outcome might differ when using raster scan images. Fourth, in the present study, 842 patients were included in the training set. Although this number is relatively larger than that reported in previous neovascular AMD studies^19–21^, it is still far smaller than the population included in studies in other fields of medicine^44^. As neovascular AMD is not a prevalent disorder, further multicenter studies are required to include a larger study population in order to improve the accuracy of the AI model. Fifth, there was no special process to reduce the noise in the OCT images. Sixth, we used AttentionGAN in our study; GAN models are often limited in their ability to handle complex translation tasks. In addition, there are other available options, such as CycleGAN and spatial GAN. Further studies will be necessary to find the optimal AI model for predicting anatomical treatment outcomes specific to each anti-VEGF agent. Finally, all the patients were Korean.

In conclusion, the GAN-based AI model demonstrated comparable or even superior performance to that of the human examiners in predicting anatomical treatment outcomes specific to each anti-VEGF agent. Although the overall accuracy is currently limited, improvement is possible through learning with more data, image enhancements such as noise control, and the implementation of AI models other than AttentionGAN.

## Data Availability

The datasets generated and/or analyzed during the current study are available from the corresponding author upon reasonable request.
